# Indirect Human Impacts Reverse Centuries of Carbon Sequestration and Salt Marsh Accretion

**DOI:** 10.1371/journal.pone.0093296

**Published:** 2014-03-27

**Authors:** Tyler C. Coverdale, Caitlin P. Brisson, Eric W. Young, Stephanie F. Yin, Jeffrey P. Donnelly, Mark D. Bertness

**Affiliations:** 1 Department of Ecology and Evolutionary Biology, Brown University, Providence, Rhode Island, United States of America; 2 Woods Hole Oceanographic Institution, Woods Hole, Massachusetts, United States of America; Bangor University, United Kingdom

## Abstract

Direct and indirect human impacts on coastal ecosystems have increased over the last several centuries, leading to unprecedented degradation of coastal habitats and loss of ecological services. Here we document a two-century temporal disparity between salt marsh accretion and subsequent loss to indirect human impacts. Field surveys, manipulative experiments and GIS analyses reveal that crab burrowing weakens the marsh peat base and facilitates further burrowing, leading to bank calving, disruption of marsh accretion, and a loss of over two centuries of sequestered carbon from the marsh edge in only three decades. Analogous temporal disparities exist in other systems and are a largely unrecognized obstacle in attaining sustainable ecosystem services in an increasingly human impacted world. In light of the growing threat of indirect impacts worldwide and despite uncertainties in the fate of lost carbon, we suggest that estimates of carbon emissions based only on direct human impacts may significantly underestimate total anthropogenic carbon emissions.

## Introduction

Human impacts on coastal ecosystems have become increasingly acute due to centuries of human activity and population growth [1]–[3]. Such impacts can be divided into direct impacts, those that affect ecosystems without passing through an ecological intermediary [4], and indirect impacts, which often unintentionally affect ecosystems as a result of unanticipated biological processes [5], [6]. Direct human impacts, such as shoreline development [7], and their effects on coastal habitat loss are well documented. However, indirect impacts including eutrophication [8], sea-level rise [9], [10] and overfishing [6] are now attracting increasing attention as novel drivers of coastal habitat loss. Understanding the relative contributions of direct and indirect impacts on ecosystem function is critical to inform conservation policies that preserve ecosystems while allowing the sustainable resource use necessary to meet the needs of growing human populations.

One of the most pressing impacts of human population growth is the acceleration of atmospheric carbon emissions from fossil fuel consumption [11]. Humans currently extract and consume sequestered carbon through petroleum, coal, and natural gas use [2], [12]. While the development of alternative energy technologies and government regulations can reduce emissions, they do little to mitigate centuries of fossil fuel consumption [13]. Understanding how human impacts affect the carbon sequestering capacity of ecosystems is a critical step in producing accurate global carbon budgets [4]. While the effects of direct human impacts (e.g. deforestation, wetlands reclamation, and agricultural conversion) can significantly reduce or even reverse natural carbon sequestering processes, the role of indirect impacts remains poorly understood. With the severity, spatial overlap and intensity of human impacts expected to increase, particularly in coastal areas [2], human activities will continue to produce unanticipated and unpredictable effects on ecological processes [14], making future carbon emissions difficult to forecast.

Carbon sequestration is an important service provided by freshwater, terrestrial and marine ecosystems [15]–[17], but coastal wetlands sequester more carbon, known as ‘blue carbon’ [4], [18], per unit area than any other habitat worldwide: 4.8–87.2 Tg C yr^−1^ globally with negligible emission of greenhouse gases [19]. Salt marsh plants remove and store atmospheric carbon through high primary productivity and flow attenuation, which leads to the deposition of waterborne organic carbon in deep peat layers. The potential of salt marshes to store carbon and reverse the effects of anthropogenic emissions has made wetland conservation an important goal worldwide [18]. However, the contribution of coastal habitat destruction to greenhouse gas emissions demonstrates the volatility of carbon sequestration in the face of human impacts [4], [20]. Furthermore, the recent inclusion of novel carbon fluxes resulting from direct habitat loss [4] significantly increased emission estimates based on loss of sequestration alone, but failed to include estimates of habitat and carbon loss from indirect impacts. Indirect impacts including eutrophication, oiling and recreational fishing, however, have recently been shown to shift this system from a carbon sink to a carbon source [5], [6], [21]. As a result, the recent estimate of 20–240 Tg C yr^−1^ [4] likely underestimates emissions from salt marshes worldwide.

Indirect human impacts occur across broad temporal scales, from years to decades, and can release carbon sequestered over centuries to millennia, making it difficult to predict how they will affect global carbon budgets. Furthermore, the fate of carbon lost from these systems is difficult to determine. While much of the carbon released from marine systems may remain *in situ*, in other coastal habitats or the deep ocean, some of this once-sequestered carbon is emitted into the atmosphere as respired CO_2_. Herein we use the term “loss” to describe the processes by which sequestered carbon is released from marshes (e.g. erosion, herbivory) and “emission” to refer to transmission of lost carbon to the atmosphere. Further investigation is needed to more accurately predict the fate of carbon lost from coastal systems, but the number of systems recently recognized as major contributors to global carbon losses suggests that direct and indirect impacts on the carbon sequestering ability of coastal systems cannot be ignored (Table 1).

**Table 1:** Direct and indirect effects of human activity on the carbon sequestering capacity of coastal ecosystems.

Ecosystem	Source of Direct Loss	Source of indirect loss
Salt marsh	Habitat conversion [4]	Eutrophication [5]
		Die-off [6], [36], [37]
		Oiling [21]
		Mosquito Ditching [24]
Seagrass/kelp forest	Boating [45]	Eutrophication [8]
	Dredging [47]	Turbidity/runoff [8]
	Destructive Fishing [50]	Sea-level rise [8]
		Trophic cascade [35]
Mangrove	Deforestation & shrimp farming [46]	Pollution [46]
		Sea-level rise [46]
Coral Reef	Dynamite Fishing [49]	Acidification [44]
	Dredging [49]	Overfishing [48]

While direct impacts receive more attention and figure prominently in carbon emission projections, indirect impacts can rival direct habitat conversion in carbon emissions and are likely to increase as coastal habitats are impacted by more intense and diverse human activities.

**Figure 1:**
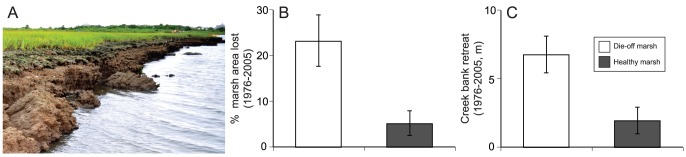
Historic salt marsh habitat loss. (A) High densities of eroded *S. reticulatum* burrows characteristic of consumer-driven die-off accelerate erosion and calving. (B) Results of GIS habitat loss analysis. Die-off marshes lost significantly more creek bank habitat between than healthy marshes between 1976 and 2005. (C) Results of GIS creek bank retreat analysis. Creek banks at die-off marshes also retreat more rapidly than healthy marsh creek banks over three decades.

**Figure 2:**
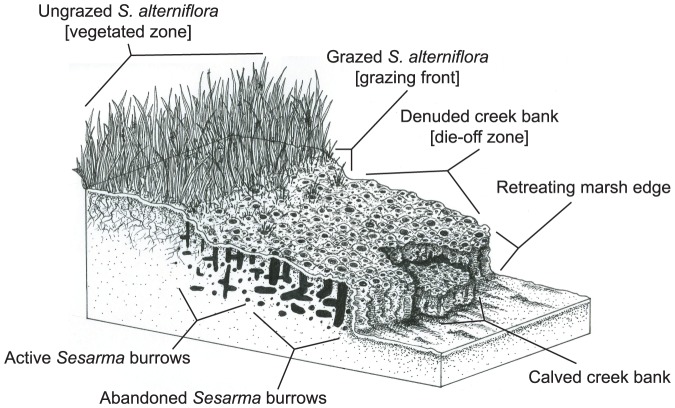
Schematic drawing of a typical die-off marsh creek bank. Experimental zones are labeled with brackets. Abandoned *S. reticulatum* burrows in the low marsh quickly collapse without maintenance, while active burrowing and herbivory create a characteristic grazing border at die-off marshes. Ungrazed *S. alterniflora* above the denuded border contributes to peat toughness, but becomes vulnerable to *S. reticulatum* grazing as the grazing front migrates into the higher marsh. (Credit: E. Suglia)

Due to their accessibility and ease of conversion, salt marshes currently face an unprecedented variety of temporally mismatched impacts [22]. One of the most pervasive contemporary indirect impacts on marshes is consumer-driven salt marsh die-off, which results from human alteration of trophic interactions [23]. New England salt marshes are the most recent example of these widespread die-offs [6], [24]. Increased herbivory by native crabs (*Sesarma reticulatum*) is driving die-off via a trophic cascade mediated by recreational overfishing. In areas along the grazing front, peat stability deteriorates and large (>1 m^3^) sections of creek bank tear from the marsh and calve into tidal creeks (Fig. 1A, 2). Here, we examine the impact of this recent trophic shift on substrate stability, habitat loss, above- and belowground carbon loss, and the accumulation of ecosystem services. Using field experiments at impacted Cape Cod marshes, geographic information system analysis of historical marsh loss, and radiocarbon dating, we demonstrate that indirect human impacts can cause previously unreported habitat loss and may contribute significantly to global anthropogenic carbon emissions.

## Methods

To quantify the extent and location of *S. reticulatum* burrowing activity, creek bank habitats at 4 sites with >30 yr histories of die-off were surveyed in July 2012. Creek bank habitat was divided into three elevation zones at each site based on vegetation characteristics: the “vegetated zone” comprised of ungrazed cordgrass, the “grazing front” characterized by conspicuous grazing scars on partially consumed cordgrass, and the completely denuded “die-off zone” characterized by dense *S. reticulatum* burrows and little remnant cordgrass following decades of overgrazing (Fig. 2). Within each zone, the number of *S. reticulatum* burrows was quantified within randomly placed 25×25 cm quadrats (n = 8 quadrats/zone) and peat toughness was measured with a penetrometer (n = 8 locations/zone). *S. reticulatum* burrows are visually distinct from fiddler crab (*Uca pugnax*) burrows in size, shape, and architecture. Percent burrow cover and peat toughness vs. elevation zone were analyzed with one-way ANOVAs. In addition, burrow density and diameter were quantified on and behind ten replicate calves and in uncalved areas at two sites. Percent burrow cover and burrow diameter were analyzed with separate one-way ANOVAs.

To investigate the effects of burrowing history on the ability of *S. reticulatum* to create and maintain burrows, we performed an overnight field burrowing trial across the three creek bank elevation zones. Single *S. reticulatum* were placed in 20×20×20 cm enclosures with access to ambient, unburrowed sediment (n = 20 enclosures/zone), and all crabs began attempting burrow creation within one hour. The size of enclosures allowed them to be placed between burrows in areas of typical burrow density. After 12 hours, burrow success was scored in each replicate: crabs that created burrows >2 cm deep were scored as successful, while those creating burrows <2 cm deep were considered unsuccessful. Previous work [25] has demonstrated that *S. reticulatum* commonly create and maintain burrows >20 cm deep in the low marsh and are vulnerable to predation and desiccation when exposed or excluded from burrows; 2 cm is sufficient for *S. reticulatum* to be avoid predation and desiccation. In addition to our general elevation surveys of peat toughness, we also measured peat toughness within 20 cm of burrows created following the above experiment (n = 20 measurements) to quantify the effects of peat toughness on burrow success. Percent burrow success and peat toughness across zones were analyzed with separate one-way ANOVAs.

To elucidate spatial patterns of creek bank erosion, sediment cores (7.5 cm diameter ×15 cm depth) from the three elevation zones were collected in August 2012 and exposed to ambient flow for one month. To mimic natural erosion exposure, each core was wrapped in 1 mm plastic tubing and capped at the bottom so that only the top surface of the core was exposed to flow. Tubes were attached to wooden stakes and installed with the tube openings 20 cm below mean high tide ∼1 m from die-off creek banks. At the conclusion of the experiment erosion rates were measured as the linear extent of erosion relative to a fixed marker on the tube (n = 3 measurements/core). Erosion rate (mm/month) vs. elevation zone was analyzed with a one-way ANOVA.

To investigate patterns of habitat loss at sites with and without *S. reticulatum*-driven die-off, low tide aerial photographs from 1976 (the putative onset of die-off on Cape Cod) and 2005 were analyzed for 8 die-off and 5 healthy (control) sites using GIS. One outlying die-off site having the greatest habitat loss and creek bank retreat was excluded from statistical analysis; the interpretation of results was not effected by its exclusion. Die-off and healthy sites were interspersed over >100 km of shoreline (Figure S1). To reduce the risk of overestimating habitat loss, only marsh habitat present in 1976 and completely submerged in 2005 was included in the analysis; areas of die-off in 2005 photographs have experienced significant vertical erosion [25], but were not included in this analysis to ensure a conservative estimate of habitat loss. The area of habitat lost between 1976 and 2005, length of creek bank habitat and total healthy marsh area were quantified for each site [24]. These data were used to calculate the proportion of total marsh area lost and the average horizontal creek bank retreat during this interval, which were analyzed with one-way ANOVA (data transformed to meet assumptions of ANOVA). In agreement with earlier analyses [6], [24], [27], we found that control sites lacking *S. reticulatum* burrows and herbivory experienced little habitat loss and creek bank retreat. As a result, we restrict further analyses of potential carbon emissions to die-off sites.

To estimate aboveground carbon loss resulting from *S. reticulatum* herbivory, ungrazed cordgrass biomass was harvested from 1 m^2^ quadrats at 9 die-off marshes (n = 8 quadrats/site) and dried to a constant weight at 60°C. Carbon sequestered in live *S. alterniflora* was estimated following drying [26]. Belowground carbon was estimated at the same 9 study sites by collecting 7.5 cm diameter cores (n = 5 cores/site), drying cores to a constant weight at 60°C and partitioning into 5 cm depth strata. The carbon content of each subsection was estimated by combusting a subsample of ∼3 grams at 550°C to remove organic carbon. The carbon lost in each subsection was multiplied by the volume of each stratum to estimate average carbon content. Both above- and belowground carbon content were scaled up to the site level using GIS analysis of aerial photographs [27]. The carbon content of *S. alterniflora* was multiplied by the area of creek bank habitat lost to estimate total aboveground carbon losses from die-off. This method likely underestimates the total loss of aboveground carbon storage capacity by assuming a one-time grazing of cordgrass without annual recovery. The volume of peat lost at each site was estimated by multiplying the area of habitat loss by an average peat depth quantified directly in August 2012 (n = 10 depths/site). Belowground carbon losses were estimated by multiplying carbon content within each strata by the volume of peat lost within that strata, with all peat loss below 20 cm assigned the carbon content of the 20-25 cm strata [28].

To determine the most likely fate of carbon lost from above- and belowground pools as a result of *S. reticulatum* activity we surveyed the recent literature for carbon conversion rates across marine ecosystems. In light of the high uncertainty of carbon conversion rates in the literature, we present reasonable upper and lower bounds for our carbon emission estimates based on a recently published global analysis [4], while also providing a brief summary of the status of this topic and discussion of current shortcomings in our understanding of carbon emissions from marine systems. Furthermore, we take several steps to ensure that our estimate of carbon loss is conservative. We exclude surface erosion in die-off creek bank areas and the ongoing loss of aboveground sequestration resulting from continued herbivore-driven die-off. We also omit significant outliers from our analyses and report predictions from upper and lower emission scenarios using the most precise range of conversion estimates available, to our knowledge. This includes an “extremely conservative” lower limit that assumes 75% local retention of lost carbon [4].

To assess the temporal disparity between the accumulation of belowground carbon and the potentially rapid loss following increases in recreational fishing pressure, we collected peat from the bottom of eroded, vertical creek banks at 3 sites in October 2012 (n = 2 samples/site). Subsamples of plant material were radiocarbon dated by organic combustion at the National Ocean Sciences AMS Facility at the Woods Hole Oceanographic Institute. This technique provides an estimate of the oldest age of accumulated peat lost to erosion, as vertical peat banks erode along their entire depth (Fig. 1A).

### Ethics Statement

All necessary permits for field sampling were obtained from the Cape Cod National Seashore.

## Results and Discussion

**Figure 3:**
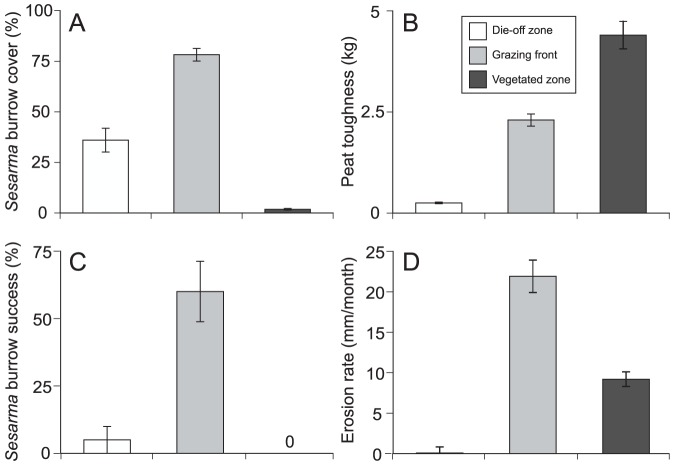
Creek bank surveys and field experiment results in three experimental zones of marsh creek banks. (A) *S. reticulatum* burrowing is largely restricted to the grazing front where substrate toughness (B) and remnant cordgrass concentrate *S. reticulatum* activity. (C) *S. reticulatum* most successfully burrow in substrate at the grazing front and are unable to start or maintain burrows in the hard vegetated zone or the soft die-off zone, respectively. (D) *S. reticulatum* burrowing and herbivory make the grazing front the site of greatest erosion, explaining patterns of creek bank calving and sediment loss.

Surveys at four die-off sites in July 2012 revealed that *S. reticulatum* burrow density is highest at the grazing front (*F*_11,84_ = 47.47, *P*<0.001, η^2^ = 0.86; Fig. 3A) and decreases in the die-off and vegetated zones. Overall, peat toughness is highest in the vegetated zone, decreases at the grazing front and is replaced by soft unconsolidated mud in the die-off zone (*F*_15,112_ = 22.46, *P*<0.001, η^2^ = 0.75; Fig. 3B). Field experiments reveal that *S. reticulatum* readily burrow at the grazing front, but are unable to burrow in the vegetated or die-off zones (*F*_2, 51_ = 18.27, *P*<0.001, η^2^ = 0.42; Fig. 3C). Peat toughness within experimental enclosures closely mirrored that in surveys across sites (*F*_2,59_ = 373.19, *P*<0.001, η^2^ = 0.93).

**Figure 4:**
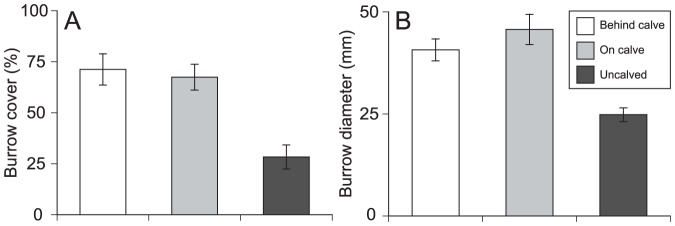
Calving surveys at die-off marshes on Cape Cod, MA. (A) *S. reticulatum* burrow density is highest on and behind calves, suggesting that burrowing weakens peat, leading to calving. Calving does not occur where burrow densities are low. (B) Burrows on and behind calves are also larger than in uncalved areas. Despite a lack of maintenance, burrows on calves erode due to tidal flow, releasing previously sequestered carbon into the water column.

Peat cores from the grazing front eroded faster than cores from the vegetated (aboveground biomass removed) and die-off zones (*F*_2,100_ = 66.65, *P*<0.001, η^2^ = 0.58; Fig. 3D). Erosion alone can result in >7.1 mm/yr of vertical peat loss at heavily burrowed sites (T. Coverdale, *unpublished data*). In addition, surveys reveal that burrow density is ∼3x higher behind and on calved sections than on nearby burrowed banks without calving (*F*_5,24_ = 14.63, *P*<0.001, η^2^ = 0.75; Fig 4A). Burrows are also larger in calved areas (*F*_5,24_ = 6.38, *P* = 0.007, η^2^ = 0.57; Fig 4B), suggesting that burrowing may weaken peat past a critical calving threshold. Calved sections lost up to 50% of their volume within one year, suggesting that carbon-rich sediment can be rapidly redistributed as a result of tidal flow (T. Coverdale, *personal observation*).

GIS analysis revealed that both habitat loss and horizontal creek bank retreat were significantly greater at die-off sites (*F*_1,11_ = 6.05, *P* = 0.032, η^2^ = 0. Fig 1B; *F*_1,11_ = 9.88, *P* = 0.009, η^2^ = 0.35; Fig 1C). Furthermore, average habitat loss and horizontal creek bank retreat were likely overestimated at healthy sites by the inclusion of one site which experienced intense storm damage between 1976 and 2005: excluding this site reduced creek bank retreat and average habitat loss at healthy sites by 40% and 60%, respectively. Across die-off sites, the area of low marsh lost from 1976 to 2005 totaled 37.8±1.6 hectares and peat depth averaged 1.49±0.1 m, resulting in a volume of creek bank habitat lost totaling 4.90×10^5^±1.97×10^4 ^m^3^. This loss represents 5.4×10^−3^±1.9×10^−4^ Gg of aboveground and 248.6±4.8 Gg of belowground carbon. Calibrated radiocarbon results indicate that the most likely age of the sediment at the current erosional surface is 18th or 19th century AD (Table 2).

**Table 2:** Radiocarbon analyses of sediment samples at the base of vertical peat faces on die-off banks.

No.	^14^C Age (ybp)	2σ calendar ages^a^	Sample Depth (m)^b^	Source
1	170±30	1660–1698 (0.209)	0.82±0.12	41.66° N, 70.03° W
2	140±30	1669–1713 (0.176)	0.82±0.12	41.66° N, 70.03° W
3	195±25	1653–1684 (0.528)	1.04±0.03	41.64° N, 70.23° W
4	160±25	1665–1699 (0.178)	1.04±0.03	41.64° N, 70.23° W
5	180±30	1653–1695 (0.196)	1.03±0.01	41.65° N, 70.18° W
6	195±20	1657–1683 (0.213)	1.03±0.01	41.65° N, 70.18° W

^a^Area under pr°bability curve [51] in parentheses

^b^site average t°tal sediment depth

Sediment lost to erosion following three decades of *S. reticulatum* herbivory and burrowing represents 150–250 years of carbon sequestration.

### Indirect Impacts and Global Threats to Coastal Carbon Sequestration

Coastal ecosystems, including salt marshes, mangroves and seagrass beds, are among the most valuable ecosystem service providers worldwide [17]. Predicted carbon emissions and warming trajectories place precedence on the management and conservation of ecosystems that remove and sequester atmospheric carbon, and globally, salt marshes sequester up to 87.2 Tg C yr^−1^, worth >$1.2 billion USD annually [18]. If preserved, salt marshes are a sustainable solution to curtailing increasing atmospheric carbon. The conversion of marshes for development, however, has historically been a primary source of wetlands loss in the United States [22], [27], and continues to be a major threat in developing nations [17], [29] with global carbon emission ramifications.

Land use change and habitat conversion are not the only human impacts that reduce the ability of salt marshes to sequester carbon. Due to increasing coastal population density and large-scale agriculture, marshes also face indirect impacts including recreational overfishing [6] and eutrophication [5], which cause loss of aboveground biomass and belowground stability. Oil spills associated with offshore drilling and shipping, such as the 2010 *Deepwater Horizon* spill, can also cause significant marsh loss through aboveground plant mortality and subsequent peat erosion [21]. These indirect impacts on salt marshes fundamentally alter ecological processes, shifting this historically bottom-up [30] ecosystem towards top-down control, increasing marsh vulnerability to consumer-driven die-off and leading to a loss of sequestered carbon [23], [31]. Similar indirect effects have been documented for other traditionally carbon-sequestering coastal habitats, where loss of habitat and services to direct impacts (e.g. land use change and reclamation) have received the bulk of attention (Table 1).

### The Ecology and Economics of Salt Marsh Die-off

Salt marsh die-off is a threat to marshes throughout the western Atlantic, where regional-scale die-offs caused by overgrazing have been expanding over the last 40 years [23]. In New England, overgrazing by *S. reticulatum* has led to conspicuous low marsh die-off and loss of marsh habitat and stored carbon (Fig. 1A). Our results revealed that substrate hardness limits *S. reticulatum* burrowing to a narrow band along the grazing front. Peat in the vegetated zone is too tough for *S. reticulatum* to create burrows and substrate in the die-off zone is too soft to support burrows. In addition to directly disturbing substrate through burrowing and increasing the surface area exposed to weathering, burrowing also increases erosion and facilitates further burrowing by weakening peat near burrow openings. Over time, communal burrows erode and widen until the peat bank passes a critical threshold and large pieces of marsh peat calve from the marsh edge and are eroded into fine sediment by tidal flow (Fig. 1A). Creek bank habitat lost to both surface erosion and calving can cause >10 cm of horizontal marsh loss annually (Coverdale unpublished data).

Aboveground cordgrass biomass in New England represents a small, labile carbon pool compared to the centuries of accumulated belowground peat lost to erosion. Substrate loss in this system also reduces creek bank elevation and cordgrass habitat, which may increase erosion rates as sea-level rises [32]–[34] further decreasing the potential for net primary productivity. Without aboveground cordgrass biomass, die-off marshes lose the ability to trap and bind sediments and annual cycles of peat accumulation are interrupted. Though tightly coupled, aboveground cordgrass biomass and belowground peat are independent carbon pools, and with consumer-driven die-off, both potentially shift from sinks to sources of atmospheric carbon [6], [17]. In areas of salt marsh die-off, sequestered carbon that was previously stored in sediments and plant biomass is released into nearshore marine habitats where it is may be stored, transported to the deep ocean, or respired. Similar estimates of carbon losses resulting from otter-induced trophic cascades [35], suggest that trophic cascades may be a widespread and underappreciated source of carbon loss from coastal ecosystems. Globally, conversion of tidal marshes alone has been estimated to contribute 20–240 Tg CO_2_ yr^−1^ to global emissions with an economic cost of 0.64–9.7 billion US$ yr^−1^ [4]. This number, however, may dramatically underestimate emissions and costs by failing to include indirect impacts.

The total area of our study sites on Cape Cod (155.7 hectares) represents only 0.003% of global salt marsh extent, but we report previously unaccounted potential carbon emissions of 2.1–8.5 Gg yr^−1^, or 0.0035–0.014% of annual emissions estimates based solely on direct human impacts. Thus, even conservative rates (25%) of conversion efficiency [4] suggest that indirect human impacts could potentially double existing estimates of carbon emissions from salt marshes worldwide. At the upper limit of conversion efficiency (100%), widespread indirect impacts, including consumer-driven die-off, oiling and eutrophication, could increase current estimates of annual anthropogenic carbon emissions from salt marshes worldwide nearly five-fold, at an additional cost of >$12.3B USD yr^−1^ [4]. Furthermore, these estimates may under-represent current regional emissions, as salt marsh die-off in New England has generally accelerated and expanded since 2005 [24]. In addition, salt marsh die-offs in the Canadian Arctic and American southeast [36], [37] have resulted in 35,000 and 100,000 hectares of habitat loss, respectively, which are not currently included in global carbon emission estimates. Salt marshes are only one of many coastal habitats currently facing unprecedented levels of direct and indirect human impacts (Table 1), which must be quantified and incorporated into future global greenhouse gas emission estimates [33]. It is important to note, however, that the above estimates reflect large uncertainty in the fate of carbon lost from coastal marshes. While some of the carbon lost from this system is surely retained *in situ*, in other nearby coastal ecosystems and/or transported to the deep ocean, a conservative 25% conversion rate still indicates a previously unaccounted for doubling in carbon emissions.

Unfortunately, high levels of uncertainty in carbon conversion estimates are widespread in marine systems. For example, recent attempts to precisely quantify carbon emissions from salt marshes, kelp forests and mangroves have been hampered by uncertainty in carbon conversion estimates, despite the fact that carbon losses were accurately quantified [4], [20], [35], [38], [39]. In these cases, the authors either present estimates covering a broad range of conversion efficiencies (as we do here) or withheld estimates entirely, citing unacceptable levels of uncertainty. This inability to accurately estimate carbon emissions is particularly striking in comparison to estimates of emissions from tropical forests: publication of emissions estimates covering a range of possible conversion rates occurred as early as 1980 [39], while multiple, latitude-specific conversion rates are common in more recent studies [40], [41]. Until the fate of marine carbon losses is similarly well understood, the contribution of marine ecosystems to global carbon budgets and future climate change scenarios will remain incomplete.

### Temporal Mismatches and the Future of Salt Marsh Ecosystem Services

Population growth and resource exploitation over the last several centuries has outstripped the ability of coastal ecosystems to provide goods and services for human populations, necessitating the development of industrialized commercial fishing, large-scale coastal infrastructure, and fossil fuel extraction. The advent of these technologies poses a far more acute threat than previous human activities in coastal wetlands [22]. Present impacts affect a larger spatial scale and create more damage over shorter time periods than those caused by earlier technologies and human population densities [7]. Contemporary impacts are also characterized by order of magnitude temporal differences between impact duration and ecological effect, where the habitat lost to years or decades of impact represents centuries or millennia of accumulated ecological processes. Here we documented such a case, where three decades of salt marsh die-off, resulting from an indirect human impact, reversed centuries of carbon sequestration. Thus, by virtue of their extent and severity, indirect human impacts have the potential to cause unintended and unanticipated changes in ecological functioning and the provisioning of ecosystem services [42].

Such rapid, large-scale impacts degrade ecosystem resilience and, with predicted increases in global population, carbon emissions, and resource consumption over the next century [2], [13], reliance on ecosystem goods and services is likely to increase. The unsustainable use of natural resources, characterized by longer recovery than extraction time, may be one of the most unrecognized hurdles in developing sustainable, affordable solutions to growing resource needs worldwide. This is particularly true in systems with long generation and recovery times, such as hardwood forests and coral reefs. Here, however, we show that even in systems with rapid recovery [43], human impacts can reverse centuries of accumulated ecosystems services. Radiocarbon dating of peat currently exposed to erosion as a result of die-off suggests that 150–250 years of peat accumulation has been reversed by only 30 years of salt marsh die-off.

Wetlands are already unable to meet the resource demands of human populations [17], [22], and future impacts will likely further diminish this capacity. Historically, the consequences of multiple human impacts on salt marshes have been difficult to predict [23], [27], [36] and projected future impacts will increase the proportion of marshes facing multiple threats. Thus, the future of salt marsh ecosystem services is uncertain.

## Supporting Information

Figure S1 **Map of Cape Cod study sites.** 14 Cape Cod, MA (USA) salt marsh study sites.
(EPS)
